# Gayborhoods as Spaces of Risk and Resilience: Associations of Gayborhood Residence with Psychological Distress and Substance Use among Ethnically Diverse Sexual Minority Men

**DOI:** 10.1007/s11524-025-00996-7

**Published:** 2025-09-15

**Authors:** Randolph C. H. Chan, Marcus Shengkai Lam, Edgar Liu, Limin Mao, Tina Gordon, Sujith Kumar Prankumar, Horas T. H. Wong

**Affiliations:** 1 Department of Social Work, The Chinese University of Hong Kong, Shatin, Hong Kong; 2 City Futures Research Centre, Faculty of Arts, Design and Architecture, University of New South Wales, Sydney, Australia; 3 Centre for Social Research in Health, University of New South Wales, Sydney, Australia; 4 New South Wales Ministry of Health, Sydney, Australia; 5 Kirby Institute, University of New South Wales, Sydney, Australia; 6 Susan Wakil School of Nursing and Midwifery, University of Sydney, Sydney, Australia

**Keywords:** Gayborhood residence, Community connectedness, Psychological distress, Alcohol use, Drug use

## Abstract

Gayborhoods are urban neighborhoods characterized by a high concentration of LGBTQ + residents, businesses, community spaces, and subcultures. Living in gayborhoods may foster a sense of community and belonging that can be particularly beneficial for sexual minority men. However, existing research on gayborhoods has predominantly centered on the experiences of White gay men. The extent to which gayborhoods serve as an inclusive space for ethnically diverse sexual minority men remains largely unexplored. This paper examines the associations of gayborhood residence with LGBTQ + community connectedness, psychological distress, and substance use among ethnically diverse sexual minority men. Utilizing data from the 2023 Gay Asian Men Survey, this paper included 1071 cisgender sexual minority men of Asian backgrounds in Australia. The results indicated that older, middle-class, and gay men were more likely to live in gayborhoods than their younger, lower-class, and bisexual counterparts. The mediation analysis revealed the coexistence of positive and negative impacts of living in gayborhoods. Specifically, gayborhood residence was positively associated with LGBTQ + community connectedness, which was in turn associated with reduced levels of psychological distress but heightened levels of alcohol and drug use. The findings have significant implications for community organizing, mental health support, and substance use prevention. While leveraging gayborhoods to foster support networks and improve mental health among Asian sexual minority men is beneficial, it is equally crucial to address the pressures associated with conforming to community norms, particularly regarding social drinking and recreational drug use.

## Introduction

Gayborhoods are neighborhoods within cities that are predominantly inhabited by LGBTQ + residents [1]. These areas often feature establishments such as bars, clubs, shops, community centers, and cultural institutions that cater primarily to LGBTQ + communities. They are known for being inclusive spaces where LGBTQ + culture and history are celebrated. Previous research has identified a range of health and psychosocial implications associated with gayborhood residence, including elevated risks such as substance use and unprotected sex, as well as protective influences such as greater involvement in the gay community and stronger community cohesion [2–5]. Nonetheless, ethnically diverse sexual minority men, especially those of Asian backgrounds, are underrepresented in these studies, rendering their experiences largely invisible. The degree to which gayborhoods are inclusive of sexual minority men from various racial and ethnic backgrounds is still largely unknown. From a sample of 1071 sexual minority men of Asian backgrounds in Australia, this paper investigates the associations of gayborhood residence with psychological distress and substance use.

### Evolution of Gayborhoods in Australia

In Australia, research on gayborhoods has largely focused on the rise, evolution, and eventual decline of Sydney’s Oxford Street. Initially, the area attracted gay men due to its relatively low cost of living, even before it became formally recognized as a gayborhood between the 1970s and 1990s [6]. However, by the early 2000s, Oxford Street began to decline, a trend attributed to gentrification [7] and the broader societal shift into the post-gay era [8, 9]. As Oxford Street declined, scholars observed the emergence of alternative “queer neighborhoods” such as Newtown in Sydney, Prahran in Melbourne, and Daylesford in regional Victoria. Unlike traditional gayborhoods, which were predominantly white and middle-class, these queer neighborhoods are more demographically diverse, encompassing a broader spectrum of gender identities, ethnicities, sexual orientations, and socioeconomic backgrounds [10]. Indeed, a small body of qualitative work has suggested that queer-friendly neighborhoods like Newtown may offer a more inclusive and welcoming environment for Asian sexual minority men compared to traditional gayborhoods like Oxford Street. Asian sexual minority men constitute a substantial and expanding segment of Australia’s gay, bisexual, and queer male population [11]. Many of them are immigrants seeking refuge from homophobia and cultural pressures related to marriage and reproduction in their countries of origin [12, 13]. Nevertheless, many Asian sexual minority men report feeling unwelcome or uncomfortable in Oxford Street due to racial segregation and fetishization [14], as well as the clash of cultural values [15]. In contrast, some of them reported feeling more at ease in queer neighborhoods such as Newtown [14].

Previous research in Australia has primarily examined gayborhood residence in the context of HIV prevention, often using postcode-level data to identify areas with higher concentrations of gay residents [16]. This approach is based on a method developed by Callander et al. [17], which has since been widely adopted in Australian studies. Several of these studies have used a threshold of 5% gay residents within a postcode to define gayborhoods [16]. Findings from this body of work have shown that postcodes with higher proportions of gay residents are associated with higher levels of PrEP uptake, more frequent HIV testing, and greater declines in HIV diagnoses [16]. While postcodes may not capture all the social and cultural nuances of gayborhoods, they serve as a meaningful proxy for areas with concentrated LGBTQ + populations and services.

### Contextual Influence of Gayborhoods

There is growing recognition that community contexts matter for health behaviors and outcomes in LGBTQ + populations [18]. Community locations such as urban or rural residence can significantly shape LGBTQ + individuals’ health behaviors, including alcohol and drug use [18]. Prior research has also shown that neighborhood characteristics such as neighborhood safety are associated with distress among sexual minority individuals [19]. Therefore, it is plausible that gayborhoods may have health implications for sexual minority men.

#### Gayborhoods as a Resource for Coping

Minority stress theory recognizes that sexual minority individuals can tap into a variety of unique resources to help buffer the detrimental impact of minority stressors [20]. Meyer [20] coined the term “minority coping” to describe group-level resources that are “related to the group’s ability to mount self-enhancing structures to counteract stigma” [20]. From this perspective, the residential clustering of LGBTQ + individuals in gayborhoods can be viewed as a group-level coping mechanism with potential mental health benefits. Specifically, Wienke et al. [21] found that sexual minority young adults who lived in gay- and queer-friendly neighborhoods had lower levels of depression symptoms and higher levels of self-esteem.

A potential pathway linking gayborhood residence to better mental health outcomes is via increased LGBTQ + community connectedness. Gayborhoods provide sexual minority individuals with resources such as shared values, increased social support and networks, as well as social and cultural events that celebrate sexual and gender diversity, all of which can contribute to a sense of pride and community. Research across different countries has demonstrated the vital role of gayborhoods in fostering LGBTQ + community connectedness and cohesion [5, 22–24]. For instance, sexual minority men residing in Sydney’s gay suburbs reported higher levels of social engagement with other sexual minority men [22]. The presence of visible LGBTQ + symbols, such as rainbow flags and pride murals [24], and pride festivals [23] reinforces a sense of belonging and identity within queer-friendly neighborhoods like Newtown, Prahran, and Daylesford in Australia. These findings align with research in the USA indicating that sexual minority men residing in gayborhoods report higher levels of community involvement and social cohesion compared to those living elsewhere [5]. Together, these studies highlight how gayborhoods serve as more than just residential spaces; they function as vital hubs that actively facilitate LGBTQ + community connectedness through both social networks and visible cultural markers. LGBTQ + community connectedness may offset feelings of isolation and distress resulting from exposure to minority stressors, contributing to better mental health outcomes [25]. Therefore, we propose that LGBTQ + community connectedness would mediate the association between gayborhood residence and psychological distress.

#### Gayborhoods as Spaces for Socializing

While gayborhoods provide spaces for LGBTQ + individuals to socialize and connect, they may also increase health-compromising behaviors [2–4]. Previous studies have explored the influence of gayborhoods on sexual minority men, but they have predominantly focused on sexual risk behaviors, such as receptive and insertive unprotected anal intercourse [2, 4, 5], with comparatively less attention given to substance use. The impact of gayborhood residence on substance use can be better understood through the lens of subcultural theory [26]. The theory posits that when individuals with similar lifestyles cluster together, they establish “a set of modal beliefs, values, norms, and customs associated with a relatively distinct social subsystem… that exists within a larger social system and culture” [26], known as a subculture. These subcultures create meaningful environments for urban residents and can significantly influence both individual and collective behaviors.

In the context of gayborhoods, the formation of LGBTQ + subcultures may shape social norms and practices, including those related to substance use. Specifically, sexual and party subcultures within gay communities in Australia have been extensively documented in the literature [27]. Social venues such as bars and nightclubs, often concentrated in gayborhoods, not only provide alcohol but also serve as settings where other substances are readily accessible through interactions with fellow patrons [3, 4]. Previous studies have shown that attendance at these social venues is associated with elevated alcohol and drug use [28], and that this association is stronger for gay licensed venues compared to other licensed venues in Australia [29]. In addition, prior research have demonstrated that gayborhood residence is associated with the use of methamphetamine and ecstasy [2, 3], as well as sexualized drug use [4]. Consequently, qualitative studies have consistently shown that substance use is often perceived as an integral aspect of gay social life [30]. For many, it serves as an entry point into the community [31] and a means of connecting with other gay men [30].

Given that substance use is deeply embedded within LGBTQ + community socialization, community connectedness may mediate the association between gayborhood residence and substance use. Previous studies have found that substance use is positively associated with gay community connectedness [32] and time spent with gay friends in Australia [33]. Additionally, Kelly et al. [4] found that possessing a gay-centric network partially mediated the association between gayborhood residence and drug use. This line of research indicates that LGBTQ + community connectedness could play a mediating role in the relationship between gayborhood residence and substance use.

### Research Objectives and Hypotheses

Grounded in minority stress theory and subcultural theory, this paper aims to (1) explore differences in demographic characteristics, LGBTQ + community connectedness, psychological distress, and substance use by gayborhood residence and (2) examine the associations of gayborhood residence with psychological distress and substance use through the mediation of LGBTQ + community connectedness. It was hypothesized that gayborhood residence would be positively associated with LGBTQ + community connectedness and substance use, but negatively associated with psychological distress (Hypothesis 1). We also hypothesized that LGBTQ + community connectedness would mediate the associations of gayborhood residence with psychological distress and substance use (Hypothesis 2).

The focus on cisgender sexual minority men in this paper, rather than the broader LGBTQ + population, is grounded in methodological and theoretical considerations. Historically, gayborhoods have predominantly evolved around cisgender gay male communities, with distinct patterns of residential concentration and community formation that differ from other LGBTQ + subgroups [6, 7, 34]. The experiences and needs of different LGBTQ + subgroups are distinct because of their unique subcultural norms and social practices [35, 36]. By focusing specifically on cisgender sexual minority men, this paper can provide more precise insights into the relationship between residential patterns, community connectedness, and health outcomes for this particular population, while acknowledging the need for parallel studies focusing on other LGBTQ + subgroups.

## Methods

### Participants and Procedure

This paper utilized data from the 2023 Gay Asian Men Survey in Australia. Recruitment was conducted through social media and email lists from partner organizations. Participants had to meet the following criteria: be at least 18 years old, identify as men of Asian heritage, have had sexual activity with men in the past 5 years, and reside in Australia at the time of the survey. After giving informed consent, participants completed the questionnaire in English, Thai, or Chinese (simplified and traditional). The study received ethics approval from the University of New South Wales, Sydney, and ACON Health, a Sydney-based LGBTQ + health and advocacy group.

A total of 1132 participants completed the survey. Sixty-one participants were excluded for being female, non-binary, or transgender, which left 1071 valid responses from cisgender sexual minority men for analysis. Their mean age was 35.65 years (SD = 10.12). The majority identified as gay (88.9%), with 6.8% identifying as bisexual and 4.3% as other (e.g., asexual, queer). 46.6% were of East Asian ethnic backgrounds, 31.7% Southeast Asian, 11.8% mixed race, 8.1% South Asian, and 1.8% other. 74.2% had lived in Australia for over 5 years, while 25.8% had lived there for less than 5 years. Most participants were citizens or permanent residents (71.9%), employed (85.2%), and had at least a Bachelor’s degree (78.3%). 36.3% of the sample had an average weekly income of $1000–$1999 in the past 6 months, followed by $2000 or more (25.0%), and below $1000 (23.2%). The median weekly earnings in Australia at the time were AUD$1300 [37]. Table 1 shows the demographic characteristics of the participants.

**Table 1:** Differences in demographic and psychosocial characteristics by gayborhood residence

	Entire sample (*N* = 1071)	Gayborhood residents (*n* = 133)	Non-gayborhood residents (*n* = 924)
	*n* (%)/mean (SD)	*n* (%)/mean (SD)	*n* (%)/mean (SD)
Age	35.65 (10.12)	37.63 (9.63)	35.41 (10.15)
Sexual orientation			
Gay	952 (88.9%)	127 (95.5%)	814 (88.1%)
Bisexual	73 (6.8%)	3 (2.3%)	69 (7.5%)
Other	46 (4.3%)	3 (2.3%)	41 (4.4%)
Ethnicity			
East Asian	499 (46.6%)	66 (49.6%)	428 (46.3%)
Southeast Asian	339 (31.7%)	39 (29.3%)	294 (31.8%)
South Asian	87 (8.1%)	13 (9.8%)	73 (7.9%)
Mixed	126 (11.8%)	12 (9.0%)	112 (12.1%)
Other	19 (1.8%)	3 (2.3%)	16 (1.7%)
Visa status			
Citizens or permanent residents	770 (71.9%)	100 (75.2%)	661 (71.5%)
Other	301 (28.1%)	33 (24.8%)	263 (28.5%)
Years living in Australia			
5 years or less	276 (25.8%)	32 (24.1%)	239 (25.9%)
More than 5 years	795 (74.2%)	101 (75.9%)	685 (74.1%)
Education			
Secondary or below	104 (9.7%)	11 (8.3%)	89 (9.6%)
Post-secondary	127 (11.9%)	16 (12.0%)	111 (12.0%)
Bachelor’s degree or higher	839 (78.3%)	106 (79.7%)	724 (78.4%)
Employment status			
Employed	913 (85.2%)	115 (86.5%)	791 (85.6%)
Unemployed	155 (14.5%)	18 (13.5%)	132 (14.3%)
Average weekly income in past 6 months			
Below $1,000	248 (23.2%)	20 (15.0%)	225 (24.4%)
$1,000-$1,999	389 (36.3%)	55 (41.4%)	331 (35.8%)
$2,000 or more	268 (25.0%)	33 (24.8%)	234 (25.3%)
Rather not disclose	166 (15.5%)	25 (18.8%)	134 (14.5%)
LGBTQ + community connectedness	2.04 (0.86)	2.34 (0.94)	2.00 (0.84)
Psychological distress	1.45 (0.95)	1.42 (0.96)	1.45 (0.95)
Alcohol use	0.99 (0.85)	1.20 (0.83)	0.97 (0.84)
Drug use			
Yes	584 (54.5%)	93 (69.9%)	484 (52.4%)
No	487 (45.5%)	40 (30.1%)	440 (47.6%)

### Measures

Participants provided their postcodes, which were then used to determine gayborhood residence. Following the approach developed by Callander et al. [17], a postcode was classified as a gayborhood if 5% or more of its residents identified as gay.

LGBTQ + community connectedness was measured by three items. Participants first indicated how connected they felt to the LGBTQ + communities in Australia. This item was rated on a 5-point Likert scale ranging from 1 (not at all connected) to 5 (very connected). The remaining items asked participants how often in the past 6 months they attended public LGBTQ + events or festivals in Australia and spent time with their LGBTQ + friends or participated in private gatherings with other LGBTQ + individuals in Australia. These items were rated on a 5-point Likert scale ranging from 1 (never) to 5 (all the time). Higher scores indicated greater levels of LGBTQ + community connectedness. The Cronbach’s alpha of the three items was 0.74 in this sample.

Psychological distress was measured using the 6-item Kessler Psychological Distress Scale (K6) [38]. Participants were asked how often they had felt nervous, hopeless, restless or fidgety, depressed, that everything was an effort, and worthless in the past 30 days. Responses were recorded on a 5-point Likert scale, ranging from 1 (none of the time) to 5 (all of the time). Higher scores represented higher levels of psychological distress. The Cronbach’s alpha of the scale was 0.92 in this sample.

Alcohol use was assessed by the 3-item Alcohol Use Disorders Identification Test (AUDIT-C) [39]. The 3 items assess the frequency of drinking, the number of alcoholic drinks on each drinking occasion, and the frequency of binge drinking episodes in the past 6 months. Each item was rated from 0 to 4, with higher scores indicating higher levels of alcohol use. The Cronbach’s alpha of the scale was 0.78 in this sample.

Drug use was assessed by asking participants which of the following substance(s) they have used in the past 6 months, including poppers, weed, meth, ecstasy, fantasy, cocaine, magic mushrooms, heroin, ketamine, and other substances. Their responses were collapsed into a dichotomous variable indicating whether they used substances or not in the past 6 months (0 = no, 1 = yes).

### Data Analysis

Logistic regression analysis was conducted to explore demographic variables (i.e., age, sexual orientation, ethnicity, visa status, years of living in Australia, education level, employment status, income level) associated with gayborhood residence. All categorical demographic variables were dummy coded for analysis. Independent samples *t*-tests were conducted to explore the differences in LGBTQ + community connectedness, psychological distress, and alcohol use between gayborhood and non-gayborhood residents. Chi-square tests were conducted to examine the association between gayborhood residence and drug use.

Structural equation modeling (SEM) was conducted to examine whether LGBTQ + community connectedness would mediate the associations of gayborhood residence with psychological distress, alcohol use, and drug use, after controlling for demographic variables (i.e., age, sexual orientation, ethnicity, visa status, years of living in Australia, education level, employment status, income level). A measurement model was first evaluated to see whether the factor structure of the variables fit the data. The latent constructs of LGBTQ + community connectedness, psychological distress, and alcohol use were indicated by their corresponding items. The model fit was determined using the following fit indices: chi-square (*χ*^2^) statistics, comparative fit index (CFI), the root mean square error of approximation (RMSEA), and the standardized root-mean square residual (SRMR). A CFI value above 0.90 indicates an acceptable model fit [40]. An RMSEA value of 0.08 or less indicates an acceptable model fit, whereas an SRMR value of 0.08 or less represents an excellent model fit [40]. The indirect effects of gayborhood residence on psychological distress, alcohol use, and drug use via LGBTQ + community connectedness were estimated using bootstrapping analysis with 1000 resamples. The significance of the indirect effects was determined using bias-corrected 95% confidence intervals (CIs) of the parameter estimates. If the 95% CI did not include zero, the indirect effects were considered significant. SPSS version 28.0 and Mplus version 8.10 were used to conduct the analyses.

## Results

### Demographic and Psychosocial Differences by Gayborhood Residence

12.4% of participants were gayborhood residents. Age was associated with greater odds of living in gayborhoods (AOR = 1.02, 95% CI [1.001, 1.04], *p* = 0.04). Bisexual men were less likely than gay men to live in gayborhoods (AOR = 0.25, 95% CI [0.08, 0.83], *p* = 0.02). Individuals who had an average weekly income of $1000–1999 (AOR = 1.83, 95% CI [1.03, 3.26], *p* = 0.04) and rather not disclose (AOR = 2.10, 95% CI [1.10, 3.99], *p* = 0.02) were more likely to live in gayborhoods than those who had an average weekly income of below $1000.

Independent samples *t*-tests revealed significant differences in LGBTQ + community connectedness [*t*(1055) = − 4.32, *p* < 0.001] and alcohol use [*t*(1055) = − 2.98, *p* = 0.003] between gayborhood residents and non-gayborhood residents, but no significant differences in psychological distress [*t*(1054) = 0.33, *p* = 0.74]. Gayborhood residents reported significantly higher levels of LGBTQ + community connectedness and alcohol use compared to non-gayborhood residents. The results of the chi-square test showed a significant association between gayborhood residence and drug use (*χ*^*2*^ = 14.44, *p* < 0.001), with a higher proportion of gayborhood residents (69.9%) having engaged in drug use in the past 6 months compared to non-gayborhood residents (52.4%).

### Associations of Gayborhood Residence with Psychological Distress and Substance Use

**Fig. 1:**
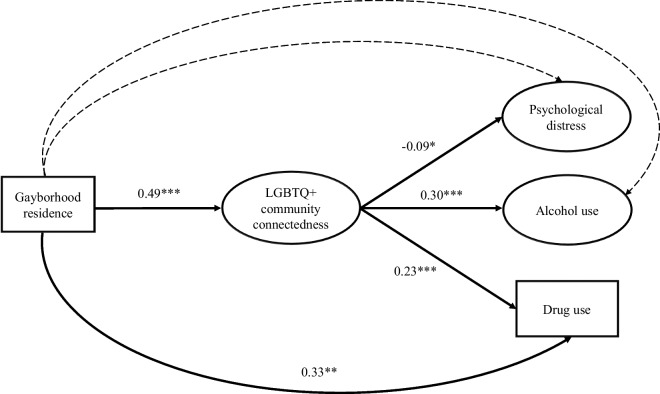
Mediation model linking gayborhood residence to psychological distress and substance use via LGBTQ + connectedness. *Notes*. *p <.05, ** *p* <.01, *** *p* <.001. Solid lines denote statistically significant paths, with standardized regression coefficients shown; dotted lines denote non-statistically significant paths. Standardized regression coefficients between 0.10 and 0.29, 0.30 and 0.49, and 0.50 or above are considered to represent small, medium, and large effect sizes, respectively. All paths are estimated while controlling for demographic variables, including age, sexual orientation, ethnicity, visa status, years living in Australia, education level, employment status, and income level

The results indicated that the measurement model showed an acceptable fit to the data, *χ*^2^ = 202.34 (*df* = 69, *p* < 0.001), CFI = 0.94, TLI = 0.92, RMSEA = 0.04, SRMR = 0.03. All loadings of the items or scales on their latent constructs (*βs* = 0.64 to 0.90) were statistically significant (*p*s < 0.001). The structural model also showed an acceptable model fit: *χ*^2^ = 380.02 (*df* = 204, *p* < 0.001), CFI = 0.94, TLI = 0.91, RMSEA = 0.03, SRMR = 0.04. As shown in Fig. 1, gayborhood residence was positively associated with LGBTQ + community connectedness (*β* = 0.49, *p* < 0.001) and drug use (*β* = 0.33, *p* = 0.007), which provides partial support to Hypothesis 1. LGBTQ + community connectedness was positively associated with alcohol use (*β* = 0.30, *p* < 0.001) and drug use (*β* = 0.23, *p* < 0.001) but negatively associated with psychological distress (*β* = − 0.09, *p* = 0.01). The unstandardized and standardized parameter estimates for the measurement and structural models are presented in Table 2.

**Table 2:** Unstandardized and standardized parameter estimates for the structural model

Parameter estimates	Unstandardized	Standardized
	B (*SE*)	*β*
Gayborhood residence → LGBTQ + community connectedness	0.38 (0.09)***	0.49***
Gayborhood residence → Psychological distress	0.06 (0.08)	0.07
Gayborhood residence → Alcohol use	0.15 (0.08)	0.20
Gayborhood residence → Drug use	0.34 (0.13)**	0.33**
LGBTQ + community connectedness → Psychological distress	− 0.10 (0.04)**	− 0.09*
LGBTQ + community connectedness → Alcohol use	0.29 (0.05)***	0.30***
LGBTQ + community connectedness → Drug use	0.31 (0.06)***	0.23***

*p <.05, **p <.01, ***p <.001. Unstandardized regression coefficients (B) indicate the expected change in the dependent variable for a one-unit increase in the independent variable, expressed in the original units of measurement; whereas standardized regression coefficients (*β*) represent the expected change in the dependent variable measured in standard deviations for a one standard deviation increase in the independent variable. Standardized regression coefficients between 0.10 and 0.29, 0.30 and 0.49, and 0.50 or above are considered to represent small, medium, and large effect sizes, respectively. All regression coefficients are estimated while controlling for demographic variables, including age, sexual orientation, ethnicity, visa status, years living in Australia, education level, employment status, and income level

A significant indirect effect was observed for gayborhood residence on psychological distress (*β* = − 0.05, 95% CI = − 0.10, − 0.01), alcohol use (*β* = 0.15, 95% CI = 0.07, 0.23), and drug use (*β* = 0.11, 95% CI = 0.05, 0.20) via LGBTQ + community connectedness. Specifically, gayborhood residence was positively associated with LGBTQ + community connectedness, which was in turn associated with reduced levels of psychological distress but heightened levels of alcohol and drug use. The findings lent firm support to Hypothesis 2.

## Discussion

As vibrant, inclusive urban spaces, gayborhoods play a significant role in shaping the social life of sexual minority men [1]. While gayborhoods are often conceived as predominately white neighborhoods [2, 5], our research found that a substantial proportion of Asian sexual minority men resided in gayborhoods in Australia. They represent various ethnic and immigration backgrounds, highlighting the existence of diverse experiences within these urban neighborhoods. Specifically, the findings revealed a distinct demographic pattern in gayborhood residence: older, gay, and middle-class men (earning $1000–1999 weekly) were more likely to inhabit these neighborhoods compared to their younger, bisexual, and lower-income counterparts (earning below $1000 weekly). This pattern aligns with previous research on white populations [2, 5], suggesting that age and income consistently influence gayborhood residence across racial groups.

The concentration of older, more affluent gay men in gayborhoods can be attributed to several interconnected historical, geographical, and social factors. In Australia, these neighborhoods are often located in inner-city areas that have undergone significant gentrification [7, 34, 41]. Many current residents established themselves in these areas decades ago, when housing costs were more affordable and before the neighborhoods became recognized as gayborhoods [6]. These places have historically been rare safe spaces where gay men could openly socialize. Over time, these early residents have developed deep roots in these neighborhoods, benefiting from established social networks and community support systems. While rising property values and gentrification have made these neighborhoods increasingly inaccessible to younger and lower-income individuals, the original residents have maintained their presence, contributing to the current demographic profile [41]. Furthermore, the transition into the post-gay era has meant that the significance of sexual identity [9] and the gay scene [8], and by extension gayborhoods, has diminished for many young sexual minority men, who now experience greater social and legal equality.

The paper provides empirical support for the contextual influence of gayborhood among Asian sexual minority men by recognizing gayborhood residence as a potential resource for coping with stress [21]. The results revealed that gayborhood residence was positively associated with LGBTQ + community connectedness, which in turn was linked to reduced psychological distress. For Asian sexual minority men, many of whom may have migrated from countries with more conservative attitudes toward sexuality, the visibility and acceptance found in Australian gayborhoods may be particularly meaningful. These neighborhoods often serve as important gateway spaces for identity exploration and community building, providing a stark contrast to familial or cultural environments that may prioritize conformity, filial piety, or heteronormative expectations [42]. Consistent with previous research [5, 24], our findings suggest that gayborhoods function not just as residential areas, but as vital support systems that contribute to the mental health of their residents.

This paper also found that Asian sexual minority men residing in gayborhoods reported higher levels of alcohol and drug use compared to their counterparts living outside gayborhoods. This may reflect the dual role of gayborhoods as both supportive environments and social spaces where substance use is more visible and, at times, normalized [2–4]. In the Australian context, where alcohol consumption is often embedded in social life, the vibrant nightlife and communal atmosphere of gayborhoods may reinforce prevailing norms around substance use. As newcomers from minority backgrounds, some Asian sexual minority men may experience unspoken social pressures to conform to substance use behaviors that are perceived as the norm, in order to gain acceptance into the prevailing (sub)culture. Additionally, the high concentration of gay bars and clubs in these areas may facilitate easier access to alcohol and recreational drugs, potentially contributing to the normalization of their use [3]. For some individuals, such as Asian migrants, who relocate to gayborhoods after experiencing hostility or exclusion in previous environments, it is also plausible that substance use serves as a coping mechanism for unresolved trauma or the ongoing stress of adapting to a new social and cultural setting [43].

Furthermore, the results showed that the associations between gayborhood residence and substance use were mediated by LGBTQ + community connectedness. This suggests that while community ties can be protective in terms of mental health, they may also expose individuals to subcultural norms that include substance use. Subcultural theory helps explain how the desire to build social connections and integrate into community life may lead individuals to adopt behaviors that are prevalent within those social circles [26]. Living in the Australian multicultural environment, Asian sexual minority men may find themselves navigating a complex interplay of cultural identities, community expectations, and social pressures [14, 44]. The visibility and accessibility of nightlife venues in gayborhoods can facilitate immersion in sexual and party subcultures [30, 31], which can be both liberating and disorienting, particularly for Asian migrants from cultures with more restrictive social norms. Within these environments, some individuals may feel compelled to conform to prevailing social practices, including more frequent substance use, as a means of gaining acceptance.

### Practical Implications

Based on the findings of this paper, there are several important implications for community organizing, mental health practice, and substance use prevention among Asian sexual minority men in Australia. Considering the positive association between gayborhood residence and LGBTQ + community connectedness, community organizing efforts should focus on fostering strong support networks and creating more inclusive spaces that cater to ethnically diverse sexual minority men. Recognizing that many LGBTQ + individuals may not feel supported in other environments, it is crucial to extend these efforts beyond gayborhoods. The development of LGBTQ-affirming spaces, such as public displays of LGBTQ + themed art, in suburban and other non-urban areas can ensure broader access to support systems and foster a sense of belonging regardless of geographical residence.

Given the observed negative association between gayborhood residence and psychological distress, mental health practitioners can leverage these neighborhoods as strategic sites for strengthening peer support and community connectedness. This could include implementing community-based therapy groups or mental health outreach programs specifically tailored to the needs of Asian sexual minority men and other underrepresented subgroups. Recognizing the influence of peer dynamics on substance use, prevention efforts might also focus on organizing substance-free social events and creating alternative spaces for connection that are not centered around alcohol or drug use. For instance, prior research in Australia has documented reductions in methamphetamine use among sexual minority men who accessed ACON’s Substance Support Service, an LGBTQ-specific alcohol and drug counseling program in New South Wales [45], as well as those who participated in the Re-Wired program, a targeted group support initiative delivered by Thorne Harbor Health in Victoria [46]. These initiatives form part of broader, multidimensional strategies that include peer-led health promotion efforts, such as the Sexually Adventurous Men project, which disseminates culturally relevant information on sexualized drug use through online platforms and collaborates with staff at sex-on-premises venues to support drug education and harm reduction [47].

Importantly, the use of postcode-level data in identifying gayborhoods has practical implications for health policy and structural interventions. This spatial framework enables policymakers and service providers to pinpoint areas where LGBTQ + populations are concentrated and may benefit most from targeted mental health and substance use resources [16, 17]. For example, resources can be strategically directed toward targeted interventions and public awareness campaigns within these identified postcodes, promoting responsible alcohol and drug use tailored to the unique needs of Asian gay communities. Moreover, this approach can inform broader strategies to ensure that such services are not limited to inner-city gayborhoods but are also extended to underserved areas. By clarifying these spatial connections, this paper underscores the value of postcode-level data in guiding equitable health resource distribution and designing interventions that are both geographically and culturally responsive.

### Limitations

Notwithstanding the contributions of this paper, a few limitations should be considered. First, the cross-sectional nature of the study prevents us from inferring causality. Longitudinal data are needed to better disentangle the relationships between gayborhood residence, psychological distress, and substance use. Second, the use of non-probability sampling methods may limit the generalizability of the findings from this paper. With the introduction of questions on sexual orientation and gender identity in the 2026 census in Australia for the first time, there will be more representative data on the demographic and psychosocial characteristics of LGBTQ + individuals living in gayborhoods. Third, while this paper offers valuable insights into the gayborhood experiences of Asian sexual minority men, it does not involve women and transgender and gender diverse individuals. Future studies should expand this work to understand the gayborhood experiences of individuals of diverse sexual orientations and gender identities. Fourth, data on participants’ migration histories were not collected. Previous experiences with LGBTQ + lifestyles in their countries of origin may influence residential preferences, particularly among those who relocated from more conservative societies. Future research should explore how migration patterns shape neighborhood choices among LGBTQ + individuals, building on existing studies of rural-to-urban migration motivated by the pursuit of more inclusive environments [34]. Fifth, we did not collect data on some neighborhood- and individual-level covariates, such as neighborhood poverty and participants’ length of residence. These factors likely influence exposure to structural and social determinants of health, and their omission limits our ability to address potential confounding effects. Future studies should incorporate these variables, such as using average taxable income to assess neighborhood socioeconomic conditions, to strengthen the validity of findings and better understand the complex relationships between neighborhood contexts and health outcomes.

## Conclusions

This paper highlights a paradox in the experiences of Asian sexual minority men living in gayborhoods in Australia: while residing in these areas is associated with stronger LGBTQ + community connectedness, which may help mitigate psychological distress, it also tends to coincide with higher levels of alcohol and drug use. These findings emphasize the importance of leveraging support networks within gayborhoods to cope with stress while recognizing the need to establish subcultures and spaces alternative to substance-centered socializing. As urban landscapes continue to evolve, understanding these dynamics in gayborhoods is crucial for creating inclusive and supportive environments for all members of LGBTQ + communities.

## Data Availability

The data used in this study are not publicly available due to concerns regarding confidentiality and data protection.
